# Efficacy of Injectable Calcium Composite Bone Substitute Augmentation for Osteoporotic Intertrochanteric Fractures: A Prospective, Non-Randomized Controlled Study

**DOI:** 10.3390/jcm14238536

**Published:** 2025-12-01

**Authors:** Chae Hun Lee, Hyoung Tae Kim, Hong Moon Sohn, Gwui Cheol Kim, Eun Ju Jin, Suenghwan Jo

**Affiliations:** 1 Department of Orthopedic Surgery, Chosun University Hospital, Gwangju 61453, Republic of Korea; 2 College of Medicine, Chosun University, Gwangju 61452, Republic of Korea; 3 TDM Co., Ltd., R&D Center, Gwangju 61003, Republic of Korea

**Keywords:** bone substitute, cephalomedullary nailing, injectable calcium-based bone substitutes, intertrochanteric fracture

## Abstract

**Background/Objectives:** Femoral intertrochanteric fractures (ITFs) in older adults are associated with a substantial risk of mechanical failure after fixation, which can lead to persistent pain, delayed mobilization, and increased mortality. Injectable calcium composite bone substitute (ICCBS) augmentation has been proposed as a strategy to enhance construct stability and promote bone healing, but clinical evidence remains limited. The purpose of this study was to evaluate the efficacy of ICCBS in the management of osteoporotic ITFs. **Methods:** We conducted a multicenter, prospective, non-randomized controlled study of patients undergoing surgical fixation for osteoporotic ITFs using proximal femoral nails. Patients who consented to augmentation received ICCBS, while the control group underwent standard fixation alone. Demographic and injury-related variables were documented, and outcome data were prospectively collected. The primary outcome was time to radiographic bone union, while secondary outcomes included functional recovery (pain and ambulatory status) and complications, including fixation failure. **Results:** The mean time to radiographic bone union did not differ significantly between groups (*p* = 0.28). However, patients receiving ICCBS augmentation reported significantly lower postoperative pain scores up to 6 weeks and demonstrated reduced lag screw sliding and varus collapse at the time of bone union. There were no significant differences in complication rates, fixation failure, or ambulatory status at last follow-up between the two groups. **Conclusions:** ICCBS augmentation may improve early postoperative pain, construct stability, and functional recovery in patients with osteoporotic ITFs, although its effect on fracture healing and long-term outcomes remains uncertain. Further high-quality randomized trials are warranted to confirm these findings.

## 1. Introduction

Femoral intertrochanteric fractures (ITFs) are common in older adults and represent a major cause of morbidity and mortality [1]. These fractures are often accompanied by medical complications and functional decline, which can ultimately shorten life expectancy [1,2]. Contemporary care pathways emphasize timely surgical fixation and early mobilization to reduce complications and accelerate recovery [2,3,4,5,6]. Despite these standards, mechanical failure after ITF fixation remains a persistent challenge, particularly in osteoporotic bone. Excessive lag screw sliding, varus collapse, and cut-out are the predominant modes of failure and may lead to early postoperative pain, delayed ambulation, and fixation failure [7,8,9].

Several technical factors that mitigate mechanical failure have been well established. The tip–apex distance (TAD) is a classic predictor of femoral head cut-out, and the integrity of the lateral femoral wall is a strong determinant of reoperation risk [10,11,12]. Nonetheless, even with meticulous surgical technique and optimal implant positioning, failures still occur in osteoporotic and unstable fractures. This has led to increasing interest in strategies that reinforce the bone–implant interface [7,13].

Injectable calcium composite bone substitutes (ICCBS), composed of calcium sulfate and calcium phosphate, have emerged as potential adjuncts to fixation in osteoporotic fractures. These materials can theoretically provide immediate structural support, are osteoconductive, and can fill voids around the implant [14,15]. Previous meta-analyses suggest that ICCBS augmentation may reduce implant migration and improve radiographic outcomes [14]. However, the effect on patient-centered outcomes, such as pain, functional recovery, and quality of life, remains uncertain [16,17]. Moreover, some studies have reported more cautious conclusions, highlighting the need for product-specific and context-specific real-world evidence [18,19].

Against this background, we conducted a multicenter study to evaluate the efficacy of ICCBS augmentation in the treatment of osteoporotic ITFs. We compared standard fixation alone with fixation augmented with ICCBS, with a focus on time to bone union, mechanical stability, and early functional recovery at predefined time points.

## 2. Materials and Methods

The study protocol was approved by the institutional review board (Chosun University Hospital, IRB No. 2024-05-010), and written informed consent was obtained from all subjects involved in the study. A multicenter, prospective, non-randomized controlled study was conducted in four hospitals on patients with osteoporotic intertrochanteric fractures (ITFs) since March 2023. During the predefined study period across participating centers, we consecutively enrolled all eligible patients who met the inclusion/exclusion criteria. This approach reflects the prospective, non-randomized design and real-world recruitment constraints.

Only patients who consented to postoperative follow-up assessment were included. Inclusion criteria were (1) patients undergoing fracture fixation surgery due to intertrochanteric fracture and (2) a diagnosis of an osteoporotic fracture, while exclusion criteria were (1) underlying or newly developed mental conditions interfering with data collection (e.g., dementia, postoperative delirium), (2) non-ambulatory status prior to the fracture, (3) underlying medical conditions or the use of medications that may interfere with bone healing (e.g., dialysis state, use of steroid), (4) reverse oblique-type fractures, and (5) an additional fracture at the ipsilateral or contralateral limb before endpoint that interferes with assessing postoperative outcomes.

All patients received a detailed explanation regarding injectable calcium composite bone substitute (ICCBS; Inject Bone, TDM, Gwangju, Republic of Korea) augmentation. ICCBS was administered only to patients who provided informed consent, and this population constitutes the case cohort, while those who did not consent formed the control cohort.

### 2.1. Surgical Technique

**Figure 1:**
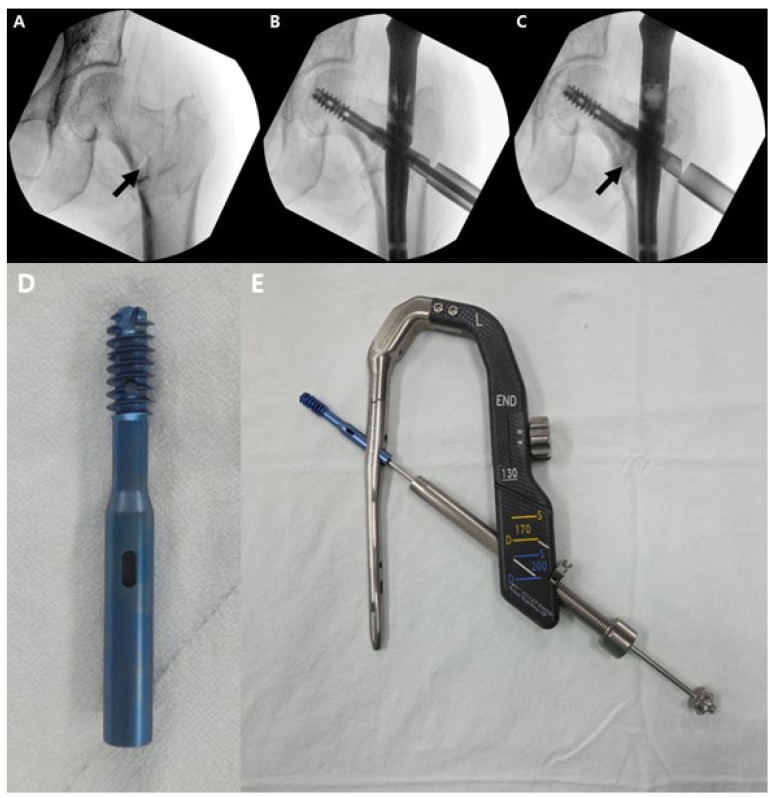
Intraoperative technique and instruments used for ICCBS augmentation during cephalomedullary nailing of intertrochanteric fractures. (**A**) Pre-injection anteroposterior fluoroscopy after reduction, showing void at reduced femoral neck region (arrow). (**B**) After lag screw insertion, and (**C**) delivery cannula docked to the lateral sleeve and injection of the calcium-based composite through the fenestrated head element; radiopaque fill within the head–neck trabecular bone is seen (arrow). (**D**) Fenestrated lag screws inferiorly permit ICCBS delivery. (**E**) Picture demonstrating the delivery system attached to the lag screw.

**Figure 2:**
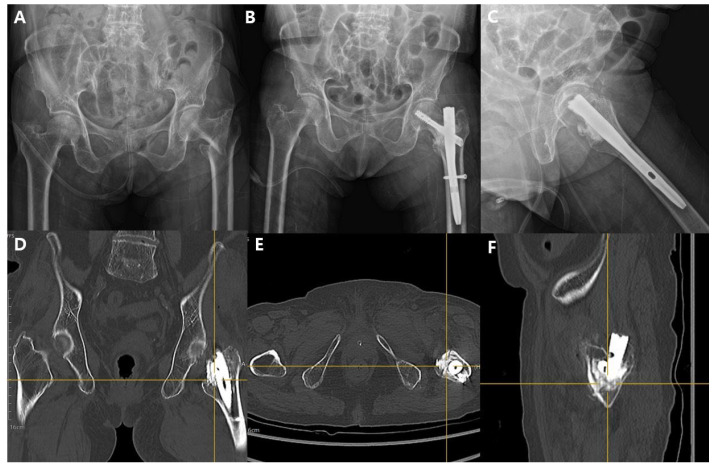
Pre- and postoperative imaging demonstrating cephalomedullary nailing with injectable calcium-based augmentation. (**A**) Preoperative anteroposterior (AP) pelvis radiograph showing an intertrochanteric fracture of the left femur. (**B**) Immediate postoperative AP pelvis view after cephalomedullary nailing. (**C**) Lateral postoperative view of the proximal femur; radiopaque material is visible along the head–neck track, consistent with ICCBS delivery via the fenestrated head element. (**D**–**F**) Multiplanar postoperative CT reconstructions—(**D**) coronal, (**E**) axial, and (**F**) sagittal planes—confirm circumferential, cloud-like distribution of the augmentation around the head element within the femoral head/neck, without intra-articular or extraosseous leakage (AP, anteroposterior; CT, computed tomography).

All operations were performed using a standard fracture table. The fracture site reduction was attempted to achieve extramedullary reduction, which was followed by insertion of a proximal femoral nail. After insertion of an appropriately sized lag screw, a delivery tube was coupled to the lag screw system, and under fluoroscopic guidance, ICCBS was injected. The lag screw system used (CHN, TDM, Gwangju, Republic of Korea) contains an approximately 8 × 5 mm hole on the distal side of the lag screw, located just medial to the cephalomedullary nail. This design allows ICCBS to be injected into the distal fragment void, which typically develops following reduction in the proximal fragment. Injection continued until the void was filled, typically requiring 6 to 10 cc (Figure 1). The control group underwent surgery using the same technique and instrumentation, but without ICCBS augmentation. The adequacy of void filling via ICCBS was confirmed through postoperative CT (Figure 2).

### 2.2. Postoperative Management

Patients were encouraged to ambulate as tolerated, beginning with wheelchair use on the first postoperative day, followed by assisted walking when possible. Full weight bearing was permitted once the patient felt safe during partial weight bearing.

### 2.3. Data Collection

Preoperative variables included demographics, pre-fracture ambulatory status, and fracture classification (AO/OTA).

Postoperatively, fracture reduction quality was assessed using the Baumgaertner criteria [20], categorized as adequate (extramedullary or anatomical) or inadequate (intramedullary). Lag screw placement was evaluated by measuring the tip–apex distance (TAD) and position according to the Cleveland index.

Serial radiographs (anteroposterior pelvis and lateral hip) were obtained immediately after surgery and at 2 weeks, at 6 weeks, at 12 weeks, and every 3 months thereafter until either bone union or implant failure occurred. At each radiographic evaluation, patient-reported outcomes were assessed using the modified Harris Hip Score (mHHS) and visual analogue scale (VAS). At the same time, ambulatory status and any complications were documented.

### 2.4. Outcome Measures

The primary outcome was bone union, defined as cortical continuity on both anteroposterior and lateral radiographs. If the fracture side union was questionable, a CT was performed for confirmation.

**Figure 3:**
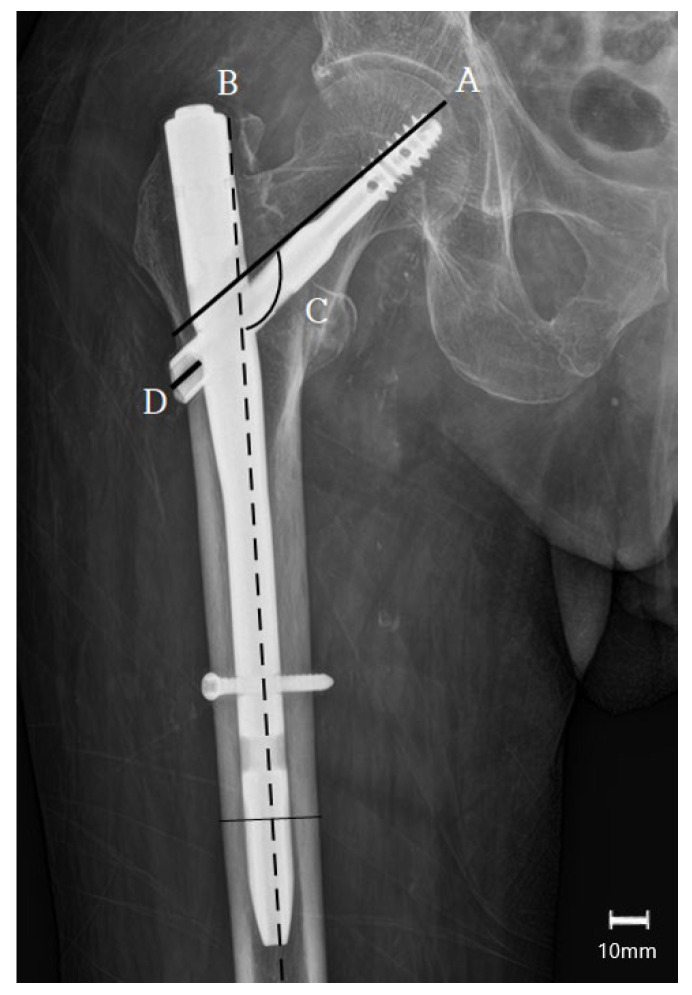
Measurement of lag screw sliding and varus collapse (ΔNSA) on anteroposterior radiographs. A: femoral neck axis; B: femoral shaft axis; C: neck–shaft angle (NSA), an angle formed by line A and B. Varus collapse is the change in NSA (angle C) between time points. Lag screw sliding is defined as the change in distance D, measured between the immediate postoperative and follow-up periods (angles are reported in degrees (°) and distances in millimeters (mm). Scale bar = 10 mm).

The secondary outcomes were a change in functional outcomes (pain and ambulatory status compared with preoperative condition) and the occurrence of complications, which include superficial or deep infection, thromboembolic events, and any adverse medical conditions. Modified Harris Hip Score (mHHS) [21,22] and visual analog scale (VAS) [23] were used to monitor functional outcome and independent pain status, while the Functional Ambulation Category (FAC) [24,25], score from 0 to 6, was used to document ambulatory status. For clinical interpretability, we set the minimal clinically important differences (MCIDs) as ~1.0–1.5 points for VAS and ~10 points for mHHS. Effects are interpreted jointly with statistical significance. Radiographic assessments included a change in the lag screw sliding distance and in the neck–shaft angle, in addition to bone union [19,26]. (Figure 3). Radiographic reduction quality was independently assessed by two fellowship-trained clinicians who did not participate in the surgeries and were blinded to group allocation and clinical outcomes. Measurements were performed on calibrated images using prespecified criteria. Significant discrepancies were resolved by consensus (and, if needed, adjudication by a third senior reviewer).

### 2.5. Statistical Assessment

Data were analyzed using IBM SPSS Statistics (ver. 20.0; IBM Corp., Armonk, NY, USA). Continuous variables are reported as mean ± SD; categorical variables as *n* (%). Between-group comparisons used independent-samples *t*-tests (with Welch’s correction if variances were unequal using Levene’s test) or Mann–Whitney U tests when the Shapiro–Wilk test indicated non-normality. Categorical outcomes (e.g., complications) were compared using Fisher’s exact or χ^2^ tests, as appropriate. Repeated outcomes (VAS at rest/mobilization, mHHS, FAC) were compared at each prespecified time point between groups; as a secondary summary, changes from the preoperative status were described. All tests were two-sided and considered significant when *p* < 0.05.

## 3. Results

One hundred sixty-four patients initially met our inclusion and exclusion criteria. However, 11 patients were unable to reach the study endpoint, defined as bone union or the development of complications such as implant failure, and were excluded from the final analysis, leaving 153 patients, which constitute the basis of our study. Of these patients, 78 were in the ICCBS augmentation group (Group A) and 75 in the non-ICCBS control group (Group B). Baseline demographics were comparable between groups and are summarized in Table 1. The mean age was 78.3 years (range: 62–89) in Group A and 76.2 years (range: 65–91) in Group B, and bone mineral density indicated osteoporosis in all patients, with comparable T-scores between the two groups. The distributions of sex and side of the fracture did not differ significantly between groups, and the preoperative ambulatory status and fracture characteristics were also similar. Surgery-related postoperative parameters were also comparable between groups in terms of reduction quality and adequacy of lag screw placement. The mean follow-up duration was 12.0 ± 3.4 months in the augmentation group and 14.0 ± 4.6 months in the control group, with no significant differences (Table 1).

Although the mean time to radiographic union in the ICCBS group was 2.2 weeks shorter than in the control group, this difference did not reach statistical significance (*p* = 0.28). Despite no significant difference in time to union between the two groups, the augmentation group showed less sliding and varus collapse, supporting mechanical stabilization rather than a biological mechanism of benefit. The mean sliding measured was 2.6 mm less, and the mean varus collapse was 4.9° less when ICCBS augmentation was utilized (Group A). Two failures occurred in group A and three in group B, all of which were converted to total hip arthroplasty. No periprosthetic fractures occurred in either group during follow-up. (Table 2).

Modified Harris Hip Score (mHHS) improved over time in both groups but remained consistently higher in the ICCBS augmentation group up to 6 weeks following the surgery. This trend was similar when the pain score was compared independently with VAS at rest and with VAS at ambulation. Ambulatory status (Functional Ambulation Category, 0–6; higher = better) was again modestly higher, with augmentation up to 2 weeks, but remained similar thereafter (Table 3). With MCID thresholds (VAS ≈1.0–1.5; mHHS ≈10), only early between-group differences were clinically meaningful. At 2 weeks, mHHS Δ = 10.7 (42.2 vs. 31.5) exceeded the MCID; VAS during mobilization also met clinical relevance when the absolute difference was ≥1.0. In contrast, VAS at rest (6 weeks Δ = 0.5) was below the MCID and likely not clinically important. Beyond 3 months, differences generally fell below the MCID and were not statistically significant, indicating convergence.

There were no signs of severe adverse medical complications; detailed counts by event type are summarized in Table 4.

## 4. Discussion

Our findings suggest that ICCBS augmentation may improve early postoperative pain, functional recovery, and construct stability in patients with osteoporotic ITFs, whereas effects on time to union and long-term outcomes remain uncertain. Although the bone-healing time was prespecified as the primary endpoint based on an initial assumption of a potential osteoconductive benefit, the observed pattern indicates that ICCBS acts predominantly via mechanical stabilization, limiting telescoping (lag screw sliding) and varus collapse, rather than accelerating callus formation. Consistent with this mechanism, time to union did not differ between groups (*p* = 0.28), while mechanical parameters and early clinical outcomes favored augmentation. In retrospect, mechanics-aligned endpoints (e.g., sliding distance, neck–shaft angle change) or early functional recovery (e.g., VAS and mHHS within 6 weeks) would likely have been more sensitive primary measures for ICCBS than time to union. Clinically, this early advantage is consequential for older adults: less pain and earlier mobility can reduce the risk of postoperative delirium, pulmonary infection, and pressure ulcers; expedite safe ambulation; and facilitate discharge planning [27]. Over time, however, biological healing and standardized rehabilitation likely diminish between-group differences as bone consolidates and patients approach functional ceilings; additional factors (e.g., analgesic titration, broad access to gait aids/physiotherapy, and selective follow-up) may further attenuate long-term contrasts.

ICCBS consists of a biphasic mixture of calcium sulfate and calcium phosphate. As a synthetic, osteoconductive, void-filling graft, it occupies metaphyseal defects and reinforces the bone–implant interface, providing front-loaded mechanical support in the early postoperative window [28]. Although robust osteo-induction has not been conclusively demonstrated for these materials, the combination of an osteoconductive scaffold and a more stable mechanical milieu may be conducive to fracture repair; on this basis, we evaluated whether ICCBS could translate interface stability into clinically meaningful gains, and our study showed promising results in terms of early pain relief and mobility recovery [29,30,31].

Numerous studies have attempted to validate whether injectable synthetic bone substitutes can provide stability to fractures. These studies observed a reduction in lag screw sliding and varus alignment when the augmented proximal femoral nail system’s fixation strength was tested under torsional and pull-out loads [19,32,33]. By occupying metaphyseal defects and reinforcing the bone–implant interface, augmentation likely reduces micromotion and toggling at the head–neck segment, thereby limiting telescoping and varus drift [7,14,19].

The clinical application of osteoconductive materials for the treatment of proximal hip fractures was tested in a previous study by Sung et al. [19], who used a zeta-potential-controlled synthetic osteoconductive graft that was intended to enhance surface interactions and cellular attachment. They reported less sliding and varus with augmentation, no difference in union time, and better Harris Hip Scores over follow-up. While there were compositional differences between the zeta-potential-controlled synthetic osteoconductive graft and ICCBS used in the current study, both materials are osteoconductive, effective in void filling, and can reinforce the bone–implant interface. The shared signal across injectable bone substitute materials suggests that interface stabilization, rather than any specific osteo-inductive effect, is the dominant driver of early clinical benefit.

**Figure 4:**
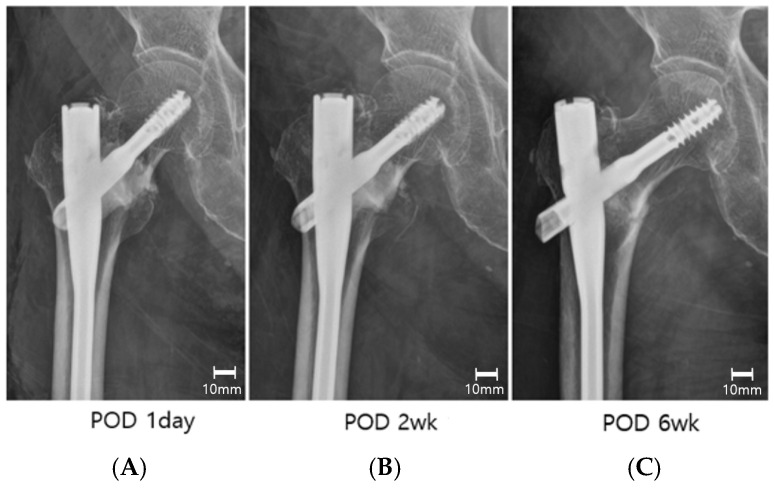
Serial AP radiographs illustrate the time course of the injectable calcium sulfate/calcium phosphate composite (ICCBS) and associated construct behavior. (**A**) POD 1 day: radiopaque ICCBS densely fills the intertrochanteric void with maintained reduction. (**B**) POD 2 weeks: conspicuous radiopacity persists, functioning as an early buttress with minimal sliding. (**C**) POD 6 weeks: ICCBS becomes faint and more sliding as remodeling proceeds (scale bar = 10 mm).

In our cohort, time to union was comparable between augmented and non-augmented fixation despite substantially less sliding and smaller varus drift in the augmentation group. This pattern is biologically coherent for osteoconductive CaSO_4_/CaP composites, which are expected to stabilize the bone–implant interface early rather than accelerate callus biology. Consistent with this, serial AP films showed conspicuous radiopacity through at least postoperative week 2, acting as an early buttress, but becoming faint or absent by 6 weeks (Figure 4), the same interval in which a between-group separation in sliding had already emerged. This time course accords with previous data showing that the calcium sulfate phase resorbs first, within weeks, allowing early vascular infiltration into the remaining calcium phosphate scaffold, whereas phosphate phases remodel over months [34]. Clinical series using injectable CaSO_4_ likewise report radiographic disappearance by 6 to 9 weeks, concordant with our imaging observations and an early, interface-stabilizing mode of action [28,35,36]. Further, updated materials-focused reviews caution that calcium sulfate-rich systems may resorb quickly and therefore require careful pressurization, leakage avoidance, and attention to defect geometry, underscoring that technique (e.g., fill-to-resistance) and case selection are as critical as the specific milliliter dose [37,38,39]. Taken together, these observations support a front-loaded benefit in the first postoperative weeks—less micromotion and less pain with mobilization—while the chronology of radiographic union remains governed by host and fracture biology [40]. Consistently, prior comparative work with a similar injectable calcium-based substitute in unstable ITF fixation reported marked reductions in sliding/varus without a clear shortening of union time, supporting the plausibility of our findings [14,19,35]. Although not statistically significant, the numerically shorter union time in the ICCBS group suggests a possible biological contribution that warrants further validation.

We acknowledge several limitations of this study. First, allocation to augmentation was non-random and based on patient consent and surgeon preference, introducing the possibility of selection bias. Although age, sex, fracture type, and BMD did not differ meaningfully between groups, the non-randomized design may limit causal inference and allow for confounding by indication, including difficult-to-measure factors (e.g., subjective bone quality, nuances of comminution). Thus, while major measured imbalances appear minimal, residual bias cannot be excluded. Second, we performed repeated between-group comparisons across outcomes and time points without multiplicity adjustment, reflecting the study’s exploratory design and constraints related to sample size and unbalanced follow-up. Consequently, Type I error inflation is possible, and nominally significant findings, particularly at later time points, should be interpreted cautiously. Third, despite the use of standardized implants and a common care pathway, center-level effects and nuances of reduction quality (e.g., anteromedial cortical support quality) may not be fully captured by our recorded covariates. Lastly, this study was not powered for rare outcomes such as cut-out and reoperation, so non-significant trends should be interpreted cautiously. Also, the durability of functional gains beyond the last follow-up of this study remains to be determined. Future studies with prespecified multivariable adjustment and randomized allocation are warranted.

## 5. Conclusions

Based on our results, injectable calcium sulfate/calcium phosphate augmentation applied to osteoporotic intertrochanteric fracture is associated with less sliding of the lag screw, smaller loss of neck–shaft angle, and superior early pain and ambulation profiles. While time to radiographic union remained similar, our findings support augmentation as a practical adjunct to standard fixation, particularly in osteoporotic bone.

## Figures and Tables

**Table 1:** Patient characteristics.

Characteristic	Group A (ICCBS Augmentation) (*n* = 78)	Group B (Non-Augmentation) (*n* = 75)	*p*-Value
Age, years (mean ± SD)	78.3 ± 8.09	76.2 ± 7.94	0.17
Sex			0.74
Male	28	24	
Female	50	51	
Side			0.80
Right	38	34	
Left	40	41	
Bone mineral density, T-score (mean ± SD)	−3.10 ± 0.99	−3.16 ± 0.70	0.67
Baumgaertner criteria			0.61
Good	41	38	
Acceptable	32	33	
Poor	5	4	
AO/OTA classification			0.56
31-A1	14	15	
31-A2	38	41	
31-A3	26	19	
Ambulatory status			0.58
Without assistance	34	32	
With assistance	44	43	
Follow-up duration, months (mean ± SD)	12 ± 3.4	14 ± 4.6	0.2

**Table 2:** Radiographic outcomes for Group A and Group B.

Outcome	Group A (*n* = 78)	Group B (*n* = 75)	*p*-Value
Time to radiographic union, weeks (mean ± SD)	11.2 ± 5.6	13.4 ± 6.2	0.28
Lagscrew sliding, mm (mean ± SD)	2.8 ± 2.1	5.4 ± 3.2	0.01 *
Varus collapse (Δ neck–shaft angle), degrees (mean ± SD)	3.3 ± 4.6	8.2 ± 5.3	0.02 *
Fixation failure	2	3	0.67

* Statistical significance (*p* < 0.05).

**Table 3:** Functional and ambulation outcomes.

Outcome /Time Point	Group A (*n* = 78)	Group B (*n* = 75)	*p*-Value
Modified Harris Hip Score (mean ± SD)			
Postoperative			
2 weeks	42.2 ± 5.34	31.52 ± 6.12	<0.001 *
6 weeks	59.3 ± 6.12	49.21 ± 4.23	0.01 *
3 months	75.2 ± 7.65	71.34 ± 8.12	0.23
6 months	77.6 ± 8.52	74.4 ± 9.56	0.31
12 months	79.6 ± 9.14	80.4 ± 7.96	0.58
Pain—VAS 0–10 at rest (mean ± SD)			
Postoperative			
2 weeks	3.2 ± 1.5	4.0 ± 1.5	0.001 *
6 weeks	1.5 ± 1.0	2.0 ± 1.2	0.02 *
3 months	1.2 ± 1.1	1.4 ± 1.0	0.24
6 months	0.7 ± 0.6	0.8 ± 0.9	0.38
12 months	0.7 ± 0.4	0.8 ± 0.5	0.55
Pain—VAS 0–10 during mobilization (mean ± SD)			
Postoperative			
2 weeks	4.5 ± 1.8	5.4 ± 1.7	<0.01 *
6 weeks	3.0 ± 1.5	3.6 ± 1.6	0.04 *
3 months	2.4 ± 1.3	2.5 ± 1.4	0.24
6 months	1.5 ± 1.2	1.8 ± 1.3	0.16
12 months	1.2 ± 1.4	1.6 ± 1.7	0.34
Ambulatory status—FAC (0–6; higher = better) (mean ± SD)			
Postoperative			
2 weeks	2.9 ± 0.9	2.1 ± 0.9	<0.001 *
6 weeks	3.5 ± 1.1	3.2 ± 1.1	0.08
3 months	4.2 ± 1.1	4.0 ± 1.2	0.27
6 months	4.6 ± 1.1	4.4 ± 1.1	0.31
12 months	4.9 ± 1.7	4.7 ± 2.1	0.60

* Statistical significance (*p* < 0.05).

**Table 4:** Postoperative complications.

Complication	Group A (*n* = 78)	Group B (*n* = 75)	*p*-Value
Superficial infection, *n*	2	1	0.58
Deep infection, *n*	0	0	-
Wound dehiscence, *n*	3	2	0.68
Urinary tract infection, *n*	7	6	0.83
Venous thrombosis, *n*	2	2	0.97
Transfusion, *n*	21	26	0.3

## Data Availability

The raw data of the current study will be made available by the authors upon request.

## Abbreviations

The following abbreviations are used in this manuscript:

ITF	Intertrochanteric fracture.
ICCBS	Injectable calcium composite bone substitute.
TAD	Tip–apex distance.
NSA	Neck shaft angle.
mHHS	Modified Harris hip score.
VAS	Visual analogue scale.
FAC	Functional ambulation category.
MCID	Minimal clinically important differences.
